# Noninvasive mobile EEG as a tool for seizure monitoring and management: A systematic review

**DOI:** 10.1111/epi.17220

**Published:** 2022-03-27

**Authors:** Andrea Biondi, Viviana Santoro, Pedro F. Viana, Petroula Laiou, Deb K. Pal, Elisa Bruno, Mark P. Richardson

**Affiliations:** ^1^ 4616 Department of Basic and Clinical Neuroscience Institute of Psychiatry, Psychology and Neuroscience King's College London London UK; ^2^ Faculty of Medicine University of Lisbon Lisbon Portugal; ^3^ 4616 Department of Biostatistics and Health Informatics Institute of Psychiatry, Psychology and Neuroscience King's College London London UK

**Keywords:** EEG, mobile, review, seizure, wearable

## Abstract

In the last two decades new noninvasive mobile electroencephalography (EEG) solutions have been developed to overcome limitations of conventional clinical EEG and to improve monitoring of patients with long‐term conditions. Despite the availability of mobile innovations, their adoption is still very limited. The aim of this study is to review the current state‐of‐the‐art and highlight the main advantages of adopting noninvasive mobile EEG solutions in clinical trials and research studies of people with epilepsy or suspected seizures. Device characteristics are described, and their evaluation is presented. Two authors independently performed a literature review in accordance with Preferred Reporting Items for Systematic Reviews and Meta‐Analyses (PRISMA) guidelines. A combination of different digital libraries was used (Embase, MEDLINE, Global Health, PsycINFO and https://clinicaltrials.gov/). Twenty‐three full‐text, six conference abstracts, and eight webpages were included, where a total of 14 noninvasive mobile solutions were identified. Published studies demonstrated at different levels how EEG recorded via mobile EEG can be used for visual detection of EEG abnormalities and for the application of automatic‐detection algorithms with acceptable specificity and sensitivity. When the quality of the signal was compared with scalp EEG, many similarities were found in the background activities and power spectrum. Several studies indicated that the experience of patients and health care providers using mobile EEG was positive in different settings. Ongoing trials are focused mostly on improving seizure‐detection accuracy and also on testing and assessing feasibility and acceptability of noninvasive devices in the hospital and at home. This review supports the potential clinical value of noninvasive mobile EEG systems and their advantages in terms of time, technical support, cost, usability, and reliability when applied to seizure detection and management. On the other hand, the limitations of the studies confirmed that future research is needed to provide more evidence regarding feasibility and acceptability in different settings, as well as the data quality and detection accuracy of new noninvasive mobile EEG solutions.

**Key Points**

- Noninvasive mobile electroencephalography (EEG) devices have been developed and are being tested to address some of the limitations of conventional scalp EEG for patients with epilepsy
- Non‐invasive mobile EEG evaluations may be well tolerated and accepted by patients with epilepsy as well as technicians and health care providers, especially because of their usability and comfort
- Available evidence suggests that EEG data collected using mobile EEG devices may be comparable to that from conventional scalp EEG, and that it can be used to visually detect EEG abnormalities and epileptic seizures with an acceptable specificity and sensitivity, and the data may be suitable for automatic‐detection algorithms
- The studies reviewed highlighted that mobile EEG has the potential to become a valuable tool in different clinical settings (ie, epilepsy monitoring unit [EMU], intensive care unit [ICU], at home, and in remote areas) to improve the diagnosis and management of people with epilepsy

## 1 INTRODUCTION

Epilepsy is characterized by an enduring predisposition to generate epileptic seizures and by neurobiological, cognitive, psychological, and social consequences.^1^ Despite epilepsy being a highly prevalent disorder, misdiagnosis is a common issue, with studies reporting a rate from 4.6% to 30%.^2^ Electroencephalography (EEG) is an important tool in the diagnosis of epilepsy,^3^ as it allows the identification of the presence of epileptiform activity, which contributes to classification and syndromic diagnosis.^4^ Long‐term recording is often required to increase the likelihood of capturing seizures or interictal activity.^3^, ^5^ The conventional approach to collecting EEG requires a long set‐up procedure, which involves skin preparation, electrode attachment, gel application, selection of montage and connection.^6^ In addition, the standard in‐hospital scalp‐EEG solution is expensive, time‐consuming, not comfortable for patients, and removes the patient from their natural environment.^6^, ^7^ Given the clinical importance of EEG findings and the limited availability of conventional EEG, there is growing interest in novel wearable or mobile EEG solutions that allow long‐term EEG monitoring in an easy‐to‐use format with acceptable performance compared to conventional EEG.^8^ Manufacturers are producing wireless EEG and dry electrodes,^9^, ^10^, ^11^ and they are reducing the number of electrodes to increase comfort and reduce the negative impact of stigma.^9^, ^12^ Despite the availability of these new EEG solutions, their adoption is still limited in clinical practice, mainly because the health care and biomedical research sectors are unfamiliar with this technology and its application.^13^

The purpose of this systematic review is to provide a detailed overview of mobile EEG innovations, and of their applications in the epilepsy health care and research settings. Specific objectives are the following: (1) to provide a comprehensive picture of the devices available, and (2) to evaluate the evidence that supports mobile EEG adoption in future clinical trials and research studies.

## 2 METHODS AND DESIGN

The systematic review was conducted according to the Preferred Reporting Items for Systematic Reviews and Meta‐Analyses (PRISMA).

### 2.1 Review inclusion criteria

#### 2.1.1 Type of technology

We included noninvasive mobile EEG systems available on the market, as well as research systems and prototypes. We focused here exclusively on mobile noninvasive devices and excluded semi‐invasive (eg, subcutaneous EEG) and fully invasive solutions (eg, intracranial implants). Subcutaneous and implanted EEG solutions in people with epilepsy have already been discussed in detail by Duun‐Henriksen et al.,^14^ Krauss et al.,^15^ and Nielsen et al.,^16^ in their comprehensive reviews.

#### 2.1.2 Type of intervention

Studies were included if an available noninvasive mobile EEG or a prototype device was tested in clinical settings (hospital, intensive care unit [ICU], ambulance) or home settings with the aim of collecting quantitative or qualitative information.

#### 2.1.3 Type of participants

Studies had to include patients with a diagnosis of epilepsy or patients suspected to have epilepsy and/or seizures requiring EEG for diagnosis. We did not apply any restriction for age, gender, ethnicity, and comorbidities.

#### 2.1.4 Type of studies

We included all original research studies (clinical studies, case‐control, case series, case report, conference abstracts). We excluded studies not available in English, reviews, book chapters, and opinion papers.

#### 2.1.5 Type of outcomes measured

We included studies where performance and experience using the devices were assessed. Information about feasibility, acceptability, tolerability, or usability collected from patients with epilepsy or health care professionals via questionnaires or interviews were included. Direct feedback from patients wearing the mobile EEG or health care professionals applying the EEG were also included. Studies describing the detection performance for EEG abnormalities (ie, seizures, epileptiform discharges, spikes) achieved by health care professionals or using automatic‐detection algorithms were included. In addition, studies comparing the quality of the recording between scalp and noninvasive mobile EEG signals (ie, background activities, number of artifacts, power spectrum analyses) were included. No meta‐analysis was planned because of the heterogeneity of the studies and outcomes.

### 2.2 Literature search

We used a three‐part search strategy to identify studies meeting the inclusion criteria above that have been published during the last 20 years (1 January 2001 to 21 January 2022): (1) electronic bibliographic databases of published works; (2) trial registers for ongoing trials; (3) a knowledge‐driven manual search online to includes other potential manuscripts, conference abstracts, devices, or ongoing trials, which can be missed by database searches. We also included relevant webpages. A protocol for this review was not registered.

### 2.3 Electronic bibliographic databases

Two authors (A.B. and V.S.) performed independently a literature review in accordance with PRISMA guidelines. A combination of different digital libraries was used (Embase, MEDLINE, Global Health, and PsycINFO). Search strategy can be found in Appendix S1. The following keyword search string was used to identify primary studies relevant to mobile EEG devices in epilepsy:

(Ear OR wireless OR Bluetooth OR portable OR mobile OR wearable OR smartphone OR rapid response) AND (EEG or electroencephalograp*) AND (epilep* OR seizur*).

Titles and abstracts of studies retrieved using the search strategy and those from additional sources were screened independently by the two authors (A.B. and V.S.) to identify studies that potentially met the inclusion criteria. Then, the full text for eligible studies was independently assessed for eligibility by the two review authors. Any disagreement over the eligibility of studies was resolved through discussion. Subsequently, ongoing clinical trials meeting the inclusion criteria described above were identified from the U.S. National Library of Medicine (https://www.clinicaltrials.gov/ ).

### 2.4 Data extraction

Two authors (A.B and V.S.) independently extracted the following relevant data from published studies on an ad hoc form: participants/population, setting, type of noninvasive mobile EEG device, aims/objectives, duration of the recording, and main results. Then, for ongoing trials, the authors extracted the following data on a second form: participants (planned to be enrolled), setting, type of noninvasive mobile EEG device, aims/objectives, and duration of the study. Noninvasive mobile EEG characteristics were finally summarized in a third form: electrode type, electrode placement, number of electrodes, sample rate, Bluetooth/wireless, seizure‐detection alarm, support needed, and battery. Discrepancies were resolved through discussion (with a third author where necessary). Due to the heterogeneity of the study characteristics and outcome measures, data synthesis and analysis was not planned or performed.

### 2.5 Quality assessment

Quality assessment was performed by authors (A.B. and V.S.) using a modified version of the Quality Assessment of Diagnostic Accuracy Studies (QUADAS‐2).^17^ It was modified to focus on the specific outcomes of the studies selected (Expert Performance, Quality of the EEG recording, Diagnostic value of mobile EEG recording in ICU, Automatic seizure detection, Usability, Tolerability, and Acceptability). Potential concerns that could affect the generalization of the presented outcomes, or the reproducibility of the study, were reported in each results section, and detailed summary tables were presented in the Appendix S1. Following previous publications^16^, ^18^ only phase‐2 or phase‐3 studies were assessed, omitting short reports or conference abstracts. An overall risk of reporting bias was not reported because of the heterogeneity of the studies.

## 3 RESULTS: 20 YEARS OF PROGRESS IN THE APPLICATION OF NEW NONINVASIVE MOBILE SOLUTIONS

**FIGURE 1:**
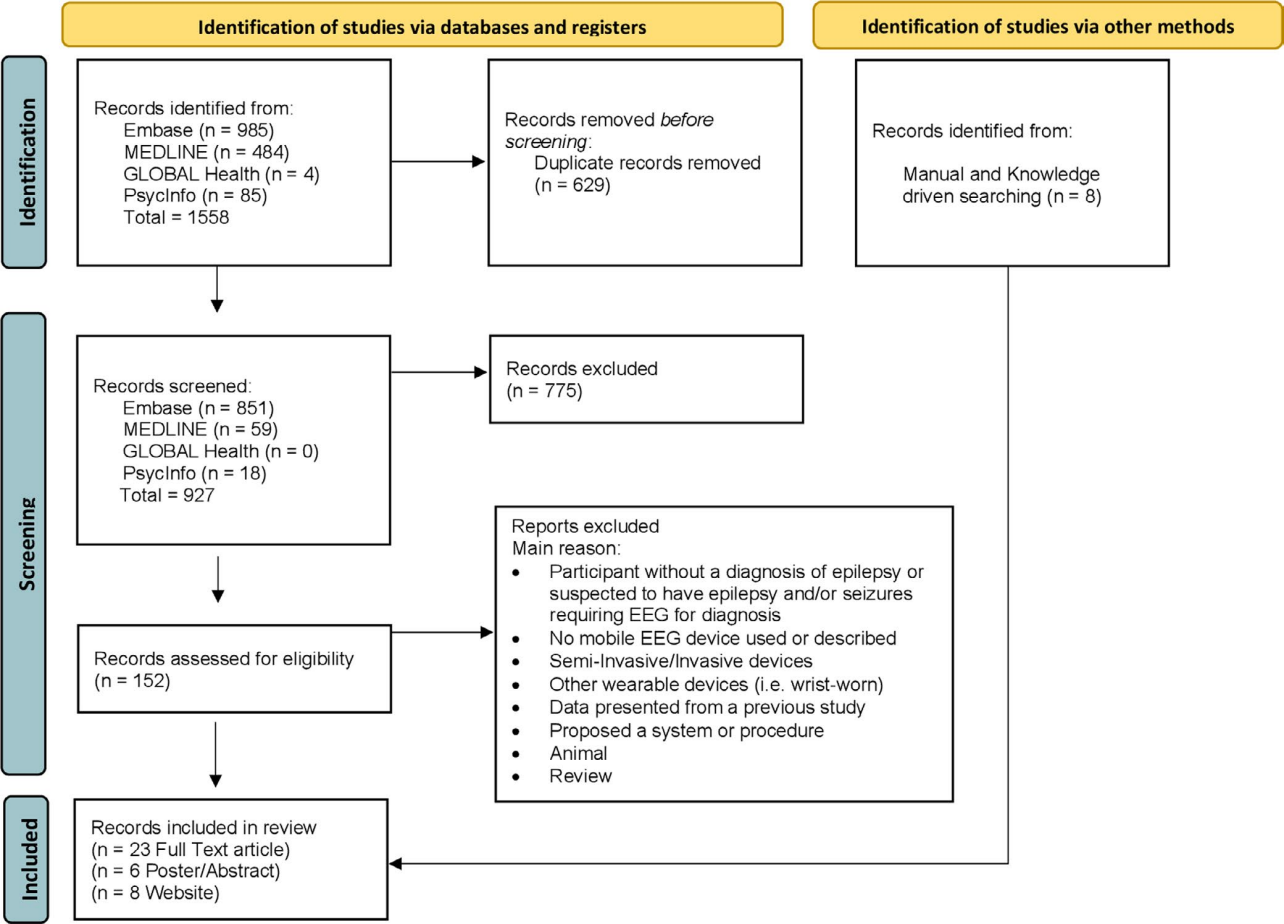
Flow diagram of the systematic review according to PRISMA guidelines

As outlined in Figure 1, the search provided a total of 927 results. Twenty‐one different studies were included from the 23 full‐text articles and 6 abstracts. Eleven studies took place in the hospital, one both in the hospital and at home, whereas three were exclusively at home, and finally seven were in the ICU or emergency department (ED). Seven studies performed 24‐h EEG studies on each participant, whereas four did not report the exact duration of the recording. Of the 21 studies, 4 presented different outcomes obtained from the same two cohorts. A total of 639 (range: 3–205; mean 65) patients with epilepsy, 21 (range: 6–15; mean 10.5) patients with suspected epilepsy, and 589 (range 5–353; mean 94.1) participants with altered mental status and suspected seizure or status epilepticus were included. Table 1 summarizes the main information from these studies.

A total of seven ongoing trials were included. Three of six will take place in the hospital, one at home, and two in both settings. A total of 1482 patients with epilepsy (range 12–750; mean 247) are planned to be enrolled. One project did not provide clear information about participant number and site. Table 2 summarizes the key information for each study.

Fourteen noninvasive mobile EEG device types were included. Nine of them were devices available on the market or US Food and Drug Administration (FDA) approved, whereas five were research prototypes. Nine mobile systems had a low number of channels (≤4), whereas six could be defined as multichannel systems. Two were available in different versions (8–64 channels), two had 14 channels, and two had 8 channels. Finally, eight devices allowed data streaming through wireless or Bluetooth and four had a seizure‐detection algorithm for automatic seizure detection. The main characteristics of the mobile solutions are summarized in Table 3.

### 3.1 Comparing performance of mobile multichannel EEG vs conventional scalp EEG in patients with epilepsy

Three studies tested multichannel EEG systems and compared the data collected with the conventional clinical EEG system. Titgemeyer et al.^19^ tested a semi‐rigid EEG headset device (Emotiv EPOC+)^20^ in the hospital epilepsy monitoring unit (EMU). Data were simultaneously collected, evaluated by 10 independent raters, and compared with respect to the presence of abnormal EEG events (regional slowing, epileptiform potentials, seizure pattern). The mobile EEG had a sensitivity of 39% and a specificity of 85% (conventional EEG 56% and 88%, respectively). They also showed that 63% of abnormalities were detected with both EEG studies, whereas 13% of abnormalities found in the conventional EEG were not present in the mobile EEG due to artifacts. Williams et al.^21^ and McKenzie et al.^22^ investigated a 14‐channel mobile, low‐cost EEG technology (SBS‐2) connected to a portable consumer‐grade amplifier and compared the data with a standard Natus EEG system in rural areas. Data were transmitted via Bluetooth connection to an Android tablet and uploaded for remote EEG specialist review and reporting via a web‐based reading platform. Williams and colleagues found that the SBS‐2 had a moderate sensitivity of 51.6% and high specificity of 90.4% for detection of epileptiform abnormalities, with positive and negative predictive values of 76.2% and 75.8%, respectively. Epileptiform discharges were detected on 25% of SBS‐2 and 37.3% of standard EEG recordings. McKenzie et al.^22^ found that the SBS‐2 had a sensitivity of 39.2% and specificity of 94.8% for detection of epileptiform discharges, and neurologists were able to identify 31% of focal and 82% of generalized abnormalities from the SBS‐2 data. Epileptiform discharges were present on 14% of SBS‐2 and 25% of standard EEG. Minor applicability concerns were related to the fact that the mobile and scalp EEG recordings did not take place sequentially so direct comparison between the performances was not allowed,^21^, ^22^ and that in some cases the agreement between experts reviewing the EEG was relatively low (<0.5).

### 3.2 Comparing performance of low channel mobile EEG vs conventional scalp EEG in patients with epilepsy

Four studies compared the detection performance for epileptiform abnormalities in devices with a low number of EEG channels. Zibrandtsen et al.^23^ compared ictal and interictal abnormalities recorded with an ear‐EEG prototype. EEG studies were compared visually by two independent neurophysiologists, and no significant difference for seizure detection was found between ear EEG and scalp EEG. Carvalho et al.^24^ developed a wearable device (Neury‐2) capable of continuously acquiring EEG from two bipolar channels. The system provided a similar spike count when compared with conventional EEG. Swinner et al.^25^ tested a mobile 4‐channel EEG called Sensor Dot (Byteflies)^26^ in patients with absence seizures. When compared with conventional EEG, blind reading of Sensor Dot data resulted in a sensitivity of 0.81 and a positive predictive value of 0.89, and an automatic seizure‐detection algorithm achieved a sensitivity of 0.83 and a positive predictive value of 0.89. Finally, Frankel et al.^27^ tested a sensor called Epilog^,^ ^28^ which allows the recording of a single channel EEG for up to 10 days. Epileptologists accurately identified seizures in 71% of Epilog recordings (84% of seizures were identified from single‐channel conventional EEG electrodes adjacent to the Epilog); convulsive seizures were more easily identified in Epilog data as compared with nonconvulsive seizures (92% vs 55%). The quality assessment of the studies presented some minor concerns because some of the devices tested were not FDA or Conformitè Europëenne marked solutions.^23^, ^24^

### 3.3 Assessing signal quality recorded via noninvasive mobile EEG in patients with epilepsy

Four studies assessed the quality of the mobile EEG signals with different methods. Zibrandtsen et al.^23^ applied correlation and time‐frequency analysis to quantify similarities between the ear‐EEG prototype and scalp EEG. Mean correlation coefficient between ear EEG and the nearest scalp electrodes was above 0.6, with a statistically significantly decreasing trend with increasing distance from the ear. Kutafina et al.^29^ compared the signal of conventional scalp EEG with the Emotiv EPOC+.^20^ Based on magnitude square coherence, the interval between 1 and 38 Hz was selected and the average Pearson correlation between the two systems on the test was 0.55 with 76% of the original signal preserved. Sokolov et al.^30^ used the SBS‐2 systems focusing on the reproducibility of EEG recordings. Their mobile EEG had an acceptable reproducibility and was useful for the detection of epileptiform discharges with an increment in diagnosis with a second EEG session of 13%. The main limitation of the study^30^ was related to the lack of video alongside the SBS‐2 EEG, which could affect the interpretation of the data recorded. Sinha, et al.^31^ tested a prototype 16‐channel EEG Cap for ambulatory use (EpiDome).^32^ The data were inspected visually by experts and then a cross‐validation of the power spectra values and the number of artifacts with the conventional EEG (for all channels and frequencies) was applied to check the quality of the recording.^31^, ^32^ They found a high cross‐validity (*R* = .897; *p* < .001) and a comparable rate of artifacts, concluding that the system was able to provide clinical‐grade EEG recording.

### 3.4 Assessing signal quality and diagnostic reliability of EEG signals recorded via noninvasive mobile EEG in the ICU and the ED

Six studies assessed the quality and the use of EEG signals recorded in the ICU and ED in patients suspected to have epilepsy and/or seizures requiring EEG for diagnosis. Meyer et al.^33^ compared the background EEG activity collected with the Rapid‐EEG by Ceribell (80channel portable solution) and the standard scalp EEG in the ICU and showed that there were no significant differences (x^2^: 7.19; *p* = .126). Furthermore, they showed that experts were able to detect ICU‐relevant EEG patterns and seizures in 89% and 98% of patients, respectively, using the CerebAir. Egawa et al.^34^ showed that neurologists were able to use the CerebAir headset to diagnose 13 patients (26%) with nonconvulsive status epilepticus (NCSE), detect NCSE with a sensitivity and specificity of 0.72 and 0.92, and detect periodic discharges (PDs) with a sensitivity of 0.82 and specificity of 0.97. Kamousi et al.^35^ assessed the signal quality of EEG waveforms acquired with an FDA‐approved 8‐channel rapid‐response EEG system (Rapid‐EEG by Ceribell^36^) on 22 patients. Multiple quality metrics were compared between Rapid‐EEG and a conventional EEG performed immediately afterwards, showing no statistical difference between all metrics except for the power of 60 Hz noise. Wright et al.^37^ similarly showed that of 38 patients wearing the Ceribell in the ICU, the one patient with NCSE was successfully diagnosed. This study also noted that physicians reported using the Rapid‐EEG contributed to changing clinical management and expedited discharge. Vespa et al.^38^ in a multicenter clinical study Does Use of Rapid Response EEG Impact Clinical Decision Making (DECIDE) trial assessed the impact on physicians’ diagnostic accuracy before and after using the Ceribell system. They found that relying on rapid response EEG information at the bedside improved the sensitivity and specificity of physicians' seizure diagnosis. Finally, Shahana et al.^39^ showed how rapid‐response EEG applied before the conventional scalp EEG can also be a useful tool helping clinicians to estimate future seizure risk compared to the 2HELPS2B. These studies presented several limitations. First of all, a mobile EEG system was not used simultaneously with the scalp EEG^34^, ^35^, ^38^; data were reviewed by different professionals with varying degrees of neurology training^38^; and participant/data selection was performed prospectively but reviewed retrospectively, which could have introduced potential selection bias.^35^, ^38^

### 3.5 Automatic‐detection algorithms applied to noninvasive mobile EEG recordings

Four studies applied or tested seizure‐detection algorithms in the data collected via mobile EEG. Kjaer et al.^40^ investigated typical absence seizures with a single‐channel mobile EEG prototype. The authors developed an automatic absence seizure‐detection algorithm based on patient‐specific modeling and achieved a sensitivity of 98.4% with 0.23 false detections per hour and a positive predictive value of 87.1%. Similarly, Swinner et al.^25^ applied a patient‐specific absence‐seizure algorithm in the Byteflies Sensor Dot data and achieved a sensitivity of 0.98 and 0.91 false detections per hour. Frankel et al.^41^ studied the accuracy of focal‐seizure detection by epileptologists, with and without the support of an automated data‐annotation algorithm applied to EEG data collected from an array of four Epilog EEG sensors attached to the scalp. They found that epileptologists, without automated data annotation, had a lower sensitivity (61%) but better false‐alarm rate (0.002/h) compared to the automated seizure‐detection algorithm (with no epileptologist involvement) that achieved a sensitivity of 90% and a false‐alarm rate of 0.087/h. Finally, Karmousi et al.^42^ evaluated a machine learning method to automatically estimate “seizure burden,” defined as the number of 10 s epochs with seizure activity in any 5 min period, with thresholds for low, medium, and high seizure burden (seizure activity in 10%, 50%, and 90% of epochs); detection of high seizure burden was used to generate a “status epilepticus” alert. EEG data were collected using the Ceribell in patients in the ICU. The machine learning algorithm had a sensitivity and specificity 100% and 93% for periods of high seizure burden; 100% and 82% for periods of medium seizure burden; and 88% and 60% for low seizure burden. Of the 179 EEG recordings in which the algorithm detected no seizures, seizures were identified by the expert reviewers in only two cases, indicating a negative predictive value of 99%. Two of the studies presented some quality concerns. Frankel and colleagues^27^ used a nonbalanced number of events for the evaluation of the diagnostic accuracy of manual seizure detection (31 epochs with ictal events and 83 nonictal), whereas Kamousi et al.^42^ pointed out that their cohort contained a relatively low number of patients with high seizure burden (9 of 353 EEG studies).

### 3.6 Usability, acceptability, and feasibility of noninvasive mobile EEG systems in patients with epilepsy

Eleven studies reported information on the acceptability of the technology. Six studies used validated questionnaires or standardized interviews and five reported direct feedback and/or adverse events reported from patients or health care professionals. Carvalho, et al.^24^ evaluated the tolerability of the Neury‐2 on 38 patients with epilepsy. Patients' experience was reported as excellent compared to long‐term ambulatory EEG. Participants did not report any concerns or interference in their daily activities using the Neury‐2. Kjaer et al.^40^ tested a single‐channel EEG prototype attached behind the ear in six children with epilepsy. The device was used for ~24 h and then the procedure was repeated after 4, 8, and 30 days. Patients and parents reported positive feedback despite feeling uncomfortable when wearing the device in public places. Similarly, Zibrandtsen et al.^23^ assessed a novel ear‐EEG prototype in 15 adults with suspected temporal lobe epilepsy. The ear EEG caused skin irritation in 13 of 15 participants. Bruno and colleagues^43^ used a modified version of the Technology Acceptance Model (TAM‐FF) questionnaire to evaluate the experience of wearing the single‐channel Epilog device (either on the forehead or behind the ear) in 12 patients undergoing conventional in‐hospital video‐EEG monitoring. The TAM‐FF indicated that the use of the technology was considered easy, although the device tended to displace during the night when attached to the forehead. Simblett, at al.^44^ interviewed a subgroup of the same patients and identified barriers to the use of this device, specifically the adhesive patch attached to the scalp, discomfort during the night, and visibility of the device. Conversely, the main facilitators of the use of the Epilog device were its practicality, its usability, and its flexibility of placement. Similarly, Olsen et al.^45^ asked nine patients with epilepsy to use a wearable EEG system with 2 channels in their home. Before and after using the device, participants were interviewed to explore their experiences. The findings illustrated that patients felt that using wearables drew attention to their epilepsy, left them feeling vulnerable, and altered their perception of themselves; hence they were less willing to use the system after a few days of monitoring. Biondi et al.^46^, ^47^ tested the acceptability and compliance of an easy‐to‐use dry EEG system (8‐channel EEG Cap Ant Neuro^48^) used by patients with epilepsy independently at home to record 10 min of eyes‐closed EEG every day for several months.^49^ The results obtained from questionnaires confirmed that the technology was well accepted after 1 month by three patients with epilepsy and that one of these patients, who completed 6 months of continuous recordings, was very satisfied with the device and achieved an optimal compliance with the daily EEG recording session. Finally, McKenzie et al.^22^ investigated the advantages of a mobile headset (SBS‐2) connected to a portable consumer‐grade amplifier in the hospital. The mobile solution was applied by medical students after <1 h of training and was well tolerated by participants and medical staff. One limitation of these studies is that they did not use standardized questionnaires or interview to assess the acceptability and usability of devices,^22^, ^23^, ^24^, ^40^ whereas one did not describe the technical characteristics of the mobile EEG tested.^45^

### 3.7 Usability, acceptability, and feasibility of noninvasive mobile EEG systems in the ICU and ED

Meyer et al.^33^ showed that one of the main advantages of using the CerebAir headset in the ICU is that it was very quick to apply due to the absence of cables and highly accepted by ICU nurses. Similarly, Egawa et al.^34^ showed that the median time needed to initiate CerebAir EEG was 57 min, saving 303 min compared to the set‐up time for conventional scalp EEG. Vespa et al.^38^ in their study assessed the timeliness and ease of use of the Rapid‐EEG Ceribell in the ICU, showing that the median time to start the Rapid‐EEG was only 5 min. The device was also rated as easy to use, and only 1 of 181 patients encountered scalp irritation. As in the previous section, only one study^38^ used a standardized scale to evaluate the usability of the noninvasive solutions, and most of the study did not report clear information about the population that was assessed.^33^, ^34^ The above studies are described further in Table 1.

**TABLE 1:** Overview of published studies

Author	Records type	Participants	Setting	EEG system	Aim	Duration	Performance and data quality	Usability and acceptability
Kjaer et al.^40^	Original manuscript	6 children with suspected epilepsy (ages 5–16)	Hospital and home	Mobile EEG recorder (Actiwave, CamNtech Ltd) connected with 3 electrodes	Evaluate how easily outpatients can be monitored with a mobile behind the ear solution. Evaluate how well an automatic seizure detection algorithm can identify absences	24 h on 4 occasions [day 1 (Hospital) while day 4, 8, 30 (Home)]	Using a patient specific model, the sensitivity for absences was 98.4% with 0.23 false detections per hour. Positive predictive value 87.1%	Patients and parents were happy and able to use the device despite feeling uncomfortable wearing it in public places
Simblett et al.^44^	Original manuscript	8 adults with a diagnosis of epilepsy^a^ ^,^ ^43^, ^44^	Hospital	Epilog	Assess the first‐hand experiences of people with epilepsy using wearable devices and understand how acceptable and easy they were to use	Mean recording 3.7 days per participant	No information provided	Barrier to use of Epilog: Adhesive patch, discomfort during night, highly visible. Facilitator to use of Epilog: Practical and simple to use, able to forget wearing it, flexible placement on head
Bruno et al.^43^	Original manuscript	12 adults with a diagnosis of epilepsy^a^ ^,^ ^43^, ^44^	Hospital	Epilog	Evaluate the experience of using wearables device during video‐EEG in patients with epilepsy	Mean recording 5.4 days. A minimum of 24 h per participant	No information provided	The TAM‐FF mean score was 3.0 ± 1.3 points, indicating that overall, the use of the technology was considered effortless. Feedback from participants described that the device tended to fall off during the night when attached on the upper forehead site. Conversely, the behind the ear position was very stable
Olsen et al.^45^	Original manuscript	9 patients with a diagnosis of epilepsy	Home	Portable EEG amplifier with 2 channels	To explore the experiences of people with epilepsy using wearables for home seizure monitoring.	Mean recording 3.5 days	No information provided	Patients felt using wearables drew attention to their epilepsy, left them feeling vulnerable, and altered their perception of themselves, hence they were less willing to use the system after a few days of monitoring
Zibrandtsen et al. ^23^	Original manuscript	15 patients with suspected temporal epilepsy	Hospital	Prototype intra‐ear EEG	Visually compare ictal and interictal abnormalities recorded with ear‐EEG and simultaneous scalp‐EEG. Quantify similarities between data collected from the two solutions	Between 1 to 4 days depending on clinical requirements	No significant differences in sensitivity and specificity for expert identification of seizures between ear‐EEG and scalp EEG data. Average Pearson correlation coefficient between ear‐EEG and the nearest scalp electrodes above 0.6	The ear‐EEG was associated with some challenges as the majority of the participant experienced some irritation linked to prolonged use of the hard earpiece (13 out of 15 participants)
Titgemeyer et al.^19^	Original manuscript	22 adults with a diagnosis of epilepsy^a^ ^,^ ^19^, ^29^	Hospital	Emotiv EPOC	Compare EEG data between a commercially available mobile EEG device and simultaneously recorded conventional scalp EEG with respect to the presence of abnormal EEG events	30 min sessions during resting state	Video EEG yielded a sensitivity of 56% and specificity of 88% while the commercial EEG showed 39% sensitivity and 88% specificity for EEG abnormalities (regional slowing, epileptiform potentials or seizure pattern)	No information provided
Sokolov et al.^30^	Original manuscript	149 patients with epilepsy	Hospital	Custom‐made mobile EasyCap with a Smartphone Brain Scanner−2 (SBS2)	Assess the quality and reproducibility of the EEG output recorded with a low‐cost mobile EEG device	Mean recording time 53 + 12.3 min (EEG1) and 29.6 + 12.8 min (EEG2)	SBS−2 had a reproducible quality level on repeated recording (EEG1 quality score 6.4 vs. EEG2 quality of 6.4) and the incremental yields of a second EEG recording of 13.2% (7 patients with ED at second diagnostic exam)	No information provided
Williams et al.^21^	Original manuscript	97 children with epilepsy (mean age 10.3)	Hospital	Custom‐made mobile EasyCap with a Smartphone Brain Scanner−2 (SBS2)	Examine a mobile, low‐cost smartphone‐based EEG technology in a heterogeneous paediatric epilepsy cohort	Mean recording time was 22.9 min	Epileptiform discharges detected on 25% of SBS−2 and 37.3% of standard EEG recording. SBS−2 had a sensitivity of 51.6% (32.4%–70.8%) and specificity of 90.4% (81.4%–94.4%) for all events. Sensitivity of 43.5% and 96.2% for generalized discharges. Positive and negative predictive value of 76.2% and 75.8% respectively for epileptiform discharges	No information provided
McKenzie et al.^22^	Original manuscript	205 patients with epilepsy	Hospital	Custom‐made mobile EasyCap with a smartphone Brain Scanner−2 (SBS2)	Assess the ability of neurologist to interpret and to detect epileptiform abnormalities from of a smartphone‐based EEG compared to standard clinical EEG	Mean recording time 30 min	Epileptiform discharges were present on 14% of SBS−2 and 25% of standard EEG. SBS−2 had a sensitivity of 39.2% (25.8% to 53.9%) and specificity of 94.8% (90.0% to 97.7%) for detection of epileptiform discharges. 31% of focal and 82% of generalized abnormalities identified with SBS−2	Both participants and medical staff did not report concerns about tolerability and usability
Sinha et al.^31^ Mukundan et al.^32^	Conference Abstract	52 patients with epilepsy	Home	Custom‐made mobile RAPIDCAP with a custom‐made visualization software	Developed an ambulatory, Hospital‐grade and user‐friendly EEG Seizure detection system (EpiDome). Compare data quality from the mobile solution and standard scalp EEG	Mean recording 30 min in resting state	Cross validation of the power spectra values and the number of artefacts between Epidome and standard scalp EEG showed high correlation (*R* = 0.897; *p* = .000) and comparable proportion of artefacts (W = 139; *p* = .432)	No information provided
Carvalho et al.^24^	Original manuscript	38 patients with continuous spike‐wave of sleep (CSWS)	Hospital	Prototype bipolar behind the ear EEG (Neury)	Demonstrate the clinical value of repeated spike index assessments using a wearable EEG device	From 24–67 h	Spike quantification from a bipolar behind the ear EEG is accurate and possible in clinical settings	The tolerability of Neury was reported as excellent by the patients, with no interference reported in their daily activities
Frankel et al.^27^	Original manuscript	40 adults with epilepsy^a^ ^,^ ^27^, ^41^	Hospital	Epilog	Determine which seizure types can be electrographically and visually counted from the mobile EEG device	Mean recording time 2.5 days	Epileptologists identified seizures in 71% of Epilog recordings and 84% of single channel wired recording adjacent to the Epilog. They achieved a 92% of accuracy identifying seizures from the Epilog data when those seizures ended in a clinical convulsion and a 55% for non‐convulsive seizures	No information provided
Frankel et al.^41^	Original manuscript	20 adults with epilepsy^a^ ^,^ ^27^, ^41^	Hospital	Epilog	Determine how accurate epileptologists are at remotely reviewing Epilog sensor EEG in the 10‐channel REMI montage” with and without seizure annotation support software. Compared with fully‐automated seizure detection algorithm	Mean recording time 2.2 days (0.5–5)	Blinded detection of focal seizures by the epileptologists, without automated data annotation, achieved a sensitivity of 61% with a mean false alarm rate of 0.002/h. With the addition of an automated data annotation algorithm, seizure detection by the epileptologists was not significantly better (68% sensitivity and false alarm rate 0.005/h)	No information provided
Swinnen et al.^25^	Original manuscript	12 adult and children with epilepsy	Hospital	Sensor Dot (Byteflies)	Investigate the performance of the Sensor Dot, to detect typical absences Develop a sensitive patient‐specific absence seizure detection algorithm to reduce the review time of the recordings	Mean recording time 24 h	Absence detection algorithm reached a sensitivity of 0.98 and false positives per hour rate of 0.91. Blind reading of full Sensor Dot data resulted in sensitivity of 0.81, positive predictive value of 0.89, and F1 score of 0.73. The review of the algorithm‐labelled files resulted in scores of 0.83, 0.89, and 0.87, respectively. The use of automated absence detection algorithm reduced the review time of a 24‐h recording from 1–2 h to around 5–10 min	No information provided
Kutafina et al.^29^	Original article	22 adults with epilepsy diagnosis^a^ ^,^ ^19^, ^29^	Hospital	Emotiv EPOC	Develop a computer‐based analysis pipeline, to compare the EEG signal acquired by a mobile EEG device to video scalp EEG	30 min long sessions in resting state	Moderate correlation between scalp EEG and portable EEG [Delta 0.62, Theta 0.73, Alpha 0.74, Beta 0.64, Full Band 0.64]	No information provided
Biondi et al.^46^	Conference Abstract	3 adults with a diagnosis of drug resistant epilepsy^a^ ^,^ ^46^, ^47^	Home	Eego amplifier‐series with 8 channels EEG Cap (ANT Neuro)	Evaluate the acceptability of a procedure that allow patients to collect independently and remotely EEG at home	Mean recording 5–10 min per day	No information provided	Total SUS score after training was 82.25 (good acceptability), while after one month the SUS was 86.37 and the overall PSSUQ score was 1.31 (high satisfaction). Average compliance for the EEG recording sessions of 86.8% (338 out of 402, 74%–98%)
Biondi et al.^47^	Conference Abstract	1 adult with a diagnosis of drug resistant epilepsy^a^ ^,^ ^46^, ^47^	Home	Eego amplifier‐series with 8 channels EEG Cap (ANT Neuro)	Describe the first experience with a long period of independently and remotely procedure that allow to record EEG independently in a patient with epilepsy	Mean recording 5–10 min per day	No information provided	Total SUS score for the EEG remained stable from the training over the end of the study (from 79 to 80). The overall PSSUQ score remained also stable (from 1.8 to 1.5). The average compliance for the EEG recording session was 88.5% (322 out of 364)
Vespa et al.^38^	Original Manuscript	164 patients with encephalopathy and suspected non‐convulsive and subclinical seizures (32% witnessed seizure)	ICU in five academic Hospital	Rapid‐EEG by Ceribell (8‐channel portable solution)	To measure the diagnosis accuracy, timeliness and easy to use of Ceribell rapid response in the ICU	Median of recording 5 min [IQR: 4–10 min]	Relying on rapid response electroencephalography information at the bedside improved the sensitivity (95% CI) of physicians’ seizure diagnosis from 77.8% (40.0%, 97.2%) to 100% (66.4%, 100%) and the specificity (95% CI) of their diagnosis from 63.9% (55.8%, 71.4%) to 89% (83.0%, 93.5%)	Median time to start Rapid‐EEG was 5 min (4–10 min) while the conventional electroencephalography was delayed by several hours (mean of 239 min). The device was rated as easy to use (mean± SD: 4.7 ± 0.6 [1 = difficult, 5 = easy]) and was without serious adverse effects
Wright et al.^37^	Short Report	38 patients with altered mental status and recent epileptic seizure or convulsive status epilepticus	Hospital emergency department (ED)	Rapid‐EEG by Ceribell (8‐channel portable solution)	Test a new bedside EEG device, Rapid Response EEG in the ED and evaluated its impact on management of suspected non‐convulsive seizure.	Not reported	The one patient with NCSE was successfully diagnosed. Physicians reported that Rapid‐EEG changed clinical management for 20 patients (53%), and expedited discharge for 8 patients (21%)	No information provided
Kamousi et al.^35^	Original Manuscript	22 patients with altered mental status and suspected nonconvulsive and subclinical seizures	Hospital Clinical ICU	Rapid‐EEG by Ceribell (8‐channel portable solution)	The purpose of this study was to address the question by evaluating the signal quality of EEG waveforms acquired with the tested rapid response EEG system in comparison to conventional clinical EEG systems in laboratory as well as clinical ICU settings	Not reported	Results confirmed that the power of 60 Hz noise in the conventional recording was higher comparing to the rapid‐EEG. The information obtained with the rapid‐EEG was concordant with the diagnostic information obtained with the conventional EEG	No information provided
Shahana et al.,^39^	Conference Abstract	5 ICU patients with clinical suspicion of seizures	ICU	Rapid‐EEG by Ceribell (8‐channel portable solution)	Comparison of rapid‐response EEG and surface EEG for seizure risk prediction using 2HELPS2B score	Not reported	Generalized or lateralized epileptiform patterns manifested in all five patients recorded with rapid‐response EEG. Based on the 2HELPS2B patients' seizure risk reflected 12%–25%. Conventional EEG immediately following rapid‐EEG confirmed the presence of electrographic seizures in three patients and NCSE in the remaining two patients	No information provided
Kamousi et al.^42^	Original Manuscript	353 adults who underwent monitoring with Rapid‐EEG Ceribell	ICU	Rapid‐EEG by Ceribell (8‐channel portable solution)	To test the performance of a machine learning method that generates bedside alerts for possible status epilepticus and measures in real time the burden of seizure activity	Not reported	The machine learning algorithm had sensitivity and specificity 100% and 93% for periods of high seizure burden; 100% and 82% for periods of medium seizure burden, and 88% and 60% for low seizure burden. Of the 179 EEG recordings in which the algorithm detected no seizures, seizures were identified by the expert reviewers in only 2 cases, indicating a negative predictive value of 99%	No information provided
Egawa et al.^34^	Original Manuscript	55 with altered mental status (6 of them [12%] with epilepsy diagnosis)	Neurointensive care unit (Neuro‐ICU)	CerebAir EEG headset (AE−120A EEG Headset)	Examine the diagnostic accuracy of Cerebair EEG monitoring in detecting abnormal EEG patterns and NCSE in patients with altered mental status (AMS) with unknown aetiology. Evaluated the time required to initiate EEG monitoring in these patients	Mean of 134.5 min in total	The sensitivity and specificity of CerebAir EEG monitoring for detecting abnormal EEG patterns were 0.97 and 0.91, respectively, for detecting PDs were 0.82 and 0.97, and for NCSE 0.7 and 0.97.2) Thirteen (26%) patients were diagnosed with NCSE using CerebAir EEG monitoring and could detect NCSE with a sensitivity and specificity of 0.706 (0.440–0.897) and 0.970 (0.842–0.999), respectively	The median time needed to initiate CerebAir EEG was 57 min (5–142) saving 303 min (219–908) needed to initiate the standard scalp‐EEG
Meyer et al.^33^	Original manuscript	52 patients with vigilance reduction ([21%] with epileptic seizure or status)	Neurointensive care unit (Neuro‐ICU)	CerebAir EEG headset	Test a novel wireless eight‐channel EEG headset developed for ICU. Compare detection performance and data quality of mobile solution and standard scalp EEG	A mean of 22.2 h of EEG	EEG background activity matched in 53% of cases (*p* = .126), seizure activity matched in 98% and epileptiform discharges in 68%. CerebAir detected in 89% of participants the same or additional relevant EEG pattern compared with standard 10/20 EEG	One of the main advantages highlighted by the authors is that the CerebAir was very quick to apply and highly accepted by ICU nurses

Information about participants, settings, non‐invasive mobile EEG, aim of the study, type of electrodes used, duration of the recording, and quantitative and qualitative results are described.

^a^ Same participants.

### 3.8 Results: Current developments with ongoing trials and projects

Seven projects are planning to test the accuracy and acceptability of new mobile EEG devices.

A validation study^50^ is running to investigate the sensitivity of EpiHunter^51^ for detection of electrographic seizures in patients with absence seizures. The system consists of a wearable single‐channel EEG combined with a video‐monitoring system. Preliminary results have been published by Loeckx et al.^52^ regarding the performance of the algorithm on scalp EEG recorded from eight patients with a total of 279 seizures.

Another trial is evaluating the Epilog^28^ in 750 patients with epilepsy. The device will be used during EMU video‐EEG recording, with the aim of determining which seizure types can be recorded and then develop a real‐time automated‐seizure alerting system for patients and caregivers.^53^

A new EEG solution called Peek is in a prototype and design phase.^54^ The device will include two electrodes that can be applied and removed behind the ear, aiming to develop continuous EEG monitoring and seizure detection. A smartphone app will also be developed to allow patients to view their results.

Another device developed during SeizeIT1 trial (2016–2019) is now being tested during the SeizeIT2 trial.^55^, ^56^ The Sensor Dot will be used on more than 500 people with refractory epilepsy who are admitted to the hospital for video‐EEG assessment. The data will be used to annotate epileptic seizures, to compare the results to the annotations made as part of routine EMU monitoring and seizure diaries kept at home, and finally to develop a seizure‐detection algorithm. The device will be also used to develop an at‐home platform named EpiCare@Home,^57^ which will allow the acquisition of multiple physiological signals, support patients at home, and provide a digital seizure diary tool.

EEG@HOME^49^ is a new project aiming to collect data from 12 adults with pharmacoresistant epilepsy, who will be asked to use a mobile EEG recording cap (ANT neuro recording system^48^) to record scalp EEG at home twice daily, wear a FitBit Charge 4, and use a smartphone app (Seer App) to collect data related to seizure occurrence. The ANT Neuro eego mini‐series (miniaturized EEG recording system) and ANT Neuro waveguard touch (8‐channel dry EEG cap) will be used at home with minimal technical support. The purpose of EEG@HOME is to develop a feasible procedure that allows people with epilepsy to acquire noninvasive biosignals independently and safely at home.

The Neuroelectrics Enobio 8^58^ mobile EEG cap was selected for the Epi Collect study.^59^ This solution was used on 50 adults with a diagnosis of epilepsy during hospitalization and at home during ambulatory monitoring and compared with scalp EEG. The collected signals will be used for developing algorithms that may identify preseizure periods and seizures. In Table 2, we summarized the key information about these projects.

**TABLE 2:** Overview of ongoing studies/trials

Title/short Title	Participants (expected to be enrolled)	Setting	Device	Aims
Ultra‐long‐term serial EEG: association of a novel seizure likelihood index with seizure occurrence, stress, sleep, and medication (EEG@HOME)^49^	12 adults with resistant epilepsy	Home	Eego amplifier‐series with 8‐ channel EEG Cap by Ant Neuro^48^	Develop a feasible procedure to collect EEG data at home independently and assess acceptability and usability of the procedure. Use the data to identify factors that increase risk of having a seizure
Clinical scenarios for long‐term monitoring of epileptic seizures with a wearable biopotential technology (SeizeIT2)^55^	500 patients (age >4 years) with refractory epilepsy	Hospital	Byteflyes Sensor Dots^26^	To annotate epileptic seizures and compare to the annotations made as part of routine EMU monitoring and seizure diaries kept at home. To develop seizure‐detection algorithms
Advanced EEG technology in childhood epilepsy (PnP)^81^	130 children (4–18 years) with refractory tonic, myoclonic or atonic seizures	Hospital and home	Byteflies Sensor Dots^26^	To study the accuracy of seizure detection in‐hospital and at home. To study the accuracy of sleep monitoring at home and hospital
Epi‐collect: data collection during video EEG monitoring and at patient's home^59^	50 adults with known diagnosis of epilepsy	Hospital and home	Enobio 8 channel mobile EEG cap by Neuroelectric^58^	To test a new mobile EEG in‐hospital and at home. To develop seizure detection algorithms
Epihunter clinical validation (ECV)^50^	40 patients (age > 4 years) with absence seizures	Hospital	Epihunter^51^	Study the sensitivity for electrographic seizures of study device compared to video EEG and self‐reported diary. Study the positive predictive value for electrographic seizures of study device compared to video EEG. To study the number of false alarms by study device per hour.
Designing a medical device for epilepsy treatment (Peek)^54^ *	N/A	N/A	A mobile behind the ear EEG device (Peek)	To develop a new mobile EEG that can be used in real time. To study the feasibility of the use of mobile devices in the hospital to collect physiological data. To use data collected to detect seizures
A Wireless EEG Patch for Continuous Electrographic Monitoring (Epilog)^53^	750 patients with previous diagnosis of Epilepsy (Age > 5 years)	Hospital	Epitel EPILOG^28^	Compare patient events noted in wired EEG against physician identified events in single channel EEG. Develop and achieve FDA clearance of an automated seizure detection system. Create a real‐time automated seizure alerting system for both a participant's personal mobile device as well as a caregiver/parent's personal mobile device. Create an hourly seizure prediction system that provides the participant with a probability of having an electrographic seizure.

Note

Information about participants, settings, non‐invasive mobile EEG, and aims of the study/trials are described in the table.

Abbreviation: N/A, information not available.

*State of the project unknown.

### 3.9 Summary of mobile EEG devices evaluated in epilepsy

Fourteen noninvasive EEG systems have been identified in this review, highlighting a strong interest in the development of portable solutions for research and clinical purposes in the field of epilepsy.

Pinho et al.^60^ and Neumann et al.^61^ suggested that an optimal mobile EEG system should meet several requirements: wireless or Bluetooth connectivity, dry electrodes, conversion with at least 24‐bit resolution, variable sampling rate, patient comfort, portability, signal artifact attenuation, event detection and prediction, and full or partial coverage of the 10–20 system for electrode placement.

Most of the new EEG solutions presented match some of these technical requirements and overcome some practical limitations of the standard method to perform a scalp EEG. In many instances, the solutions may be more comfortable and easier to set up compared to the standard scalp EEG,^20^, ^36^, ^49^, ^58^ whereas the low visibility and patient‐centered design of the device can alleviate the negative impact and social stigma of a highly visible monitoring device.^19^, ^21^, ^27^, ^49^, ^52^, ^62^ In fact, some solutions are very small and can be covered by hair or simply removed when needed.^24^, ^28^, ^36^, ^51^ Some can be also used for short‐term,^36^, ^62^ repeated,^30^ or long‐term recording with minimal support.^20^, ^49^, ^58^ Furthermore, most of the devices allow the data to be automatically streamed in real time using Bluetooth or Wi‐Fi,^20^, ^28^, ^36^, ^51^, ^62^, ^63^, ^64^ stored on a secure server,^24^, ^31^, ^32^, ^63^ and shared with the clinical specialist. Finally, the cost of the systems may be lower than conventional scalp EEG in the hospital, and availability is not limited by the availability of hospital facilities and trained technicians.^21^, ^30^, ^31^, ^32^, ^36^ Despite the advantages highlighted, researchers and clinicians need to take into account whether the technical characteristics of the devices have been carefully evaluated in relation to the purpose, the population, and the settings in which they will be used. In most instances, a robust real‐world validation has not yet been carried out. In Table 3 we summarized the technical specifications of the mobile EEG systems presented.

**TABLE 3:** Summary of technical characteristics of mobile EEG devices

Mobile EEG System	Electrodes	Battery	Sample rate	Number of Channels	Electrodes Placement	Resolution	Wireless/Bluetooth data transmission	Seizure detection algorithm	Support for the application or use of the system
Sensor Dot (SD, Byteflies, Antwerpen, Belgium)^26^	Removable electrodes attached by disposable patches	Rechargeable (up to 24 h)	Up to 256 Hz	Up to 4	Behind each ear (but other configurations are possible)	24 bits	No	No	Support needed to attach the active EEG electrodes on the scalp. Expert and non‐expert can be trained to apply it
Custom made mobile EasyCap (combination with Smartphone Brain Scanner−2 (SBS2))^30^	Ring electrodes (Gel)	Rechargeable (up to 12 h)	Up to 128 Hz	14	10–20 system	24 bits	Yes	No	Expert and non‐expert can be trained to apply it (<1 h training)
Epoc+ (EMOTIV, San Francisco, California, USA)^20^	Saline based electrodes	Rechargeable (up to 12 h)	128 to 256 Hz	14	10–20 system	16 bits	Yes	No	Expert and non‐expert can be trained to apply it (4–5 min to apply it)
CerebAir EEG headset and amplifier (Nihon Kohden Europe, Rosbach, Germany)^64^	Pre‐coated gel electrodes attached by a push button at specific positions of the headset	Rechargeable	N/A	8	10–20 system	N/A	Yes	Yes	Expert and non‐expert can be trained to apply it
Epilog (Epitel Biotechnology, Salt Lake City, Utah, USA)^28^	Removable electrodes attached by adhesive patch	Rechargeable (up to 7 days)	Up to 512 Hz	1	Behind ear or on forehead	24 bits	Yes	Yes	Minimal support – patient can be independent
EpiHunter (EpiHunter NV, Hasselt, Belgium)^51^	Three gold‐plated frontal copper dry sensors	Rechargeable (up to 4 h)	N/A	3	Electrodes mounted on a Velcro strip and removable head band	N/A	Yes	Yes	Minimal support – patient can be independent
Eego amplifier‐series with 8 channels EEG Cap (Ant Neuro, Hengelo, Netherlands)^48^	Dry silver electrodes	Powered via connection with a computer	Up to 2084 Hz	8 up to 64	10–20 system	Up to 24 bits	no	No	Expert and non‐expert can be trained to apply it (<1 h training)
Enobio EEG (Neuroelectric, Barcelona, Spain)^58^	gel or dry electrode solutions available	Rechargeable (operating life of 5.5 h with wireless data transmission)	Up to 125 Hz	8 up to 32	10–20 System	24 bits	Yes	No	Expert and non‐expert can be trained to apply it (<1 h training)
Wireless behind the ear‐EEG protorype^63^	Silver/silver chloride wet gel electrodes	Rechargeable battery (± 6.5 h)	256 Hz	2	Behind each ear (but flexible position, other configurations are possible)	12 bits	Yes	No	Expert and non‐expert can be trained to apply it
Intra‐ear‐EEG prototype^23^	Four wet in‐the‐ear recording electrodes embedded in an earpiece	Powered via connection to an external amplifier.	256 or 1024 Hz	4	Specific positions within the external auditory canal	N/A	No	No	Support needed to place gel in the active EEG electrodes
Mobile single channel EEG prototype^40^	Three electrodes (Ambu Neuroline 700 Denmark)	Powered via connection to an external amplifier.	128 Hz	1	Specific position: one attached on Fp1 (Reference), one on F7 (Active1) and one on TP7 (Active2)	N/A	No	No	Support needed to place the active EEG electrodes. Patients can be trained to fix electrodes if needed
Neury, a mobile EEG prototype^24^	Standard disk electrodes	Powered via connection to an external amplifier.	Up to 200 Hz	2	Electrodes can be placed in flexible positions	N/A	No	No	Support needed to place the EEG electrodes.
Rapid‐EEG portable EEG headband by Ceribell (Mountain View, CA)^36^	Elastic band that contains 10 Ag/AgCl electrodes (19.8 mm). Conductive gel is needed	Powered by an external recorder (Ceribell Model C100)	Up to 250 Hz. Frequency range from 0.5 to 100 Hz	Up to 8	Circumferential 10‐electrode montage‐ Corresponding approximately to the Fp1–F7, F7–T3, T3–T5, and T5–O1 sites on the left and the Fp2–F8, F8–T4, T4–T6, and T6–O2 sites on the right	N/A	Yes	Yes	Expert and non‐expert can be trained to apply it
Prototype of an ear transparent EEG – cEEGrids^62^	Flexprint material placed around the ear and held on the skin with an adhesive. Conductive part of electrodes made using Ag/AgCl	Powered by an amplifier located at the back of the head (Smarting from https://mbraintrain.com)	Up to 500 Hz	Up to 10	A total of 10 electrodes arranged in a C‐shape around the ear. Channels on the left: L1, L2, L3, L4, L4A, LAB, L5, L6, L7, L8. Channels on the right: R1, R2, R3, R4, R4A, R4B, R5, R6, R7, R8	24 bits	Yes	No	Expert and non‐expert can be trained to apply it

Abbreviation: N/A information not available.

## 4 DISCUSSION

**FIGURE 2:**
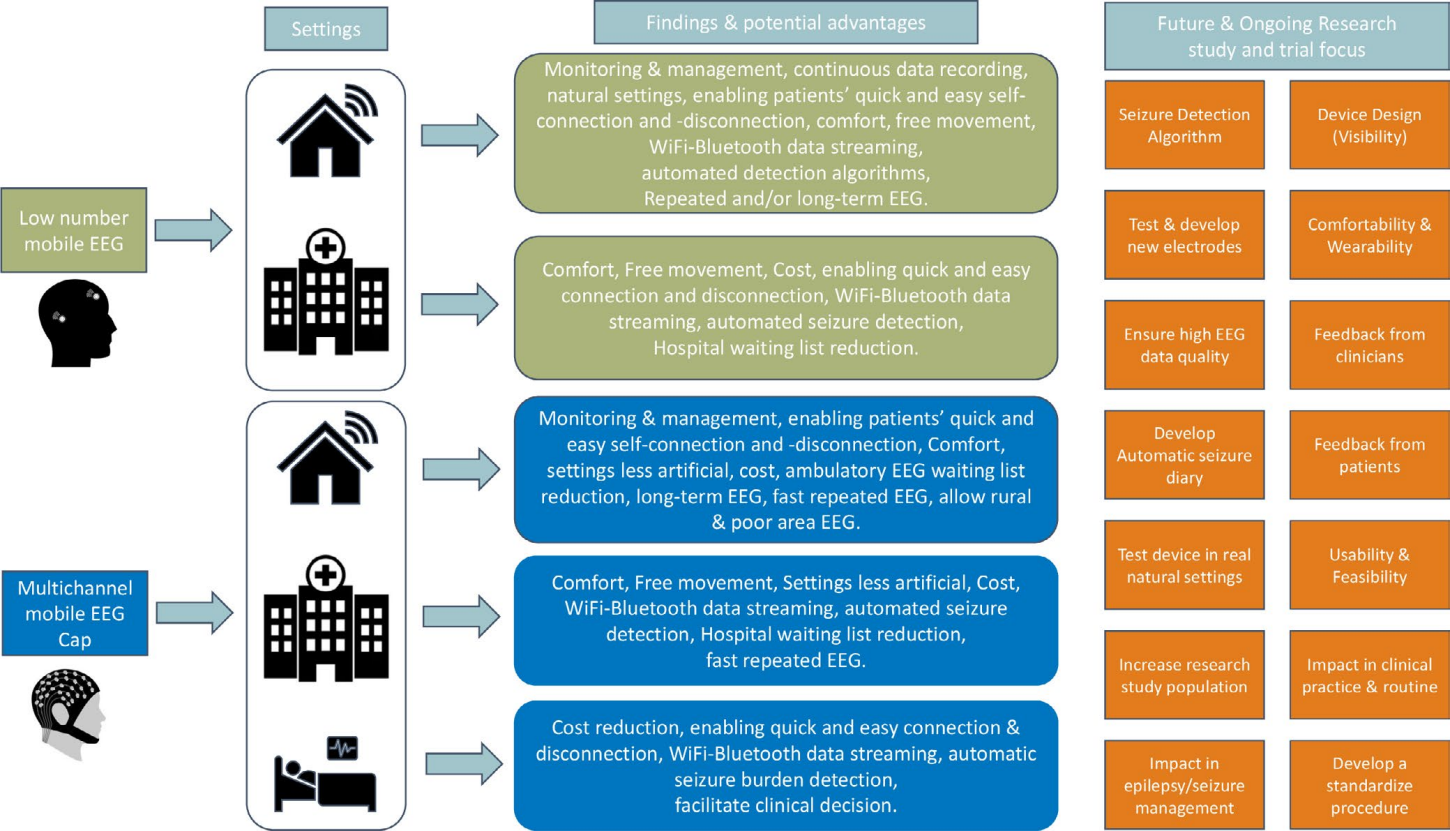
From left to right. Findings and advantages of low number (light green) and multichannel (blue) non‐Invasive Mobile EEG as tools for seizure monitoring and management. On the right of the figure key factors (orange) that need to be addressed in ongoing and future studies to increase the possibility that non‐invasive solutions will be applied in clinical practice or patients' daily life

The aim of this review was to comprehensively summarize the current literature on noninvasive mobile EEG for seizure monitoring and management. Figure 2 presents the main findings and factors that need to be addressed in the future regarding the use of mobile noninvasive solutions.

The main limitations of the review are related to the heterogeneity of the studies reported. Different devices, settings, and methods were used, and heterogeneous outcomes were presented between studies. Despite this, reviewed studies suggest that new mobile EEG systems provide data with a quality comparable with conventional scalp EEG^10^, ^19^, ^21^, ^22^, ^23^, ^24^, ^27^, ^29^, ^31^, ^33^ and can be used in multiple settings (EMU,^19^, ^23^, ^24^, ^27^, ^63^ ICU,^20^, ^24^, ^25^, ^28^, ^30^, ^33^, ^49^, ^64^ home^21^, ^40^, ^49^, ^59^). EEG data from multiple‐channel EEG showed promising results for visual detection of abnormal epileptiform events^19^, ^30^ and for different clinical purposes in different clinical settings^33^, ^34^ or rural areas,^21^, ^22^ whereas low‐channel solutions provided promising results regarding the possibility of visually detecting abnormal EEG patterns^23^, ^24^, ^25^, ^41^, ^65^ in the EMU and, when paired with seizure‐detection algorithms,^25^, ^27^, ^29^, ^40^, ^52^, ^65^, ^66^ some of these devices detected seizures with an overall acceptable sensitivity and specificity,^27^, ^41^ especially absences.^25^

Overall, evidence showed that mobile EEG is well accepted and tolerated by patients^20^, ^24^, ^40^, ^43^, ^44^, ^45^, ^46^, ^47^, ^49^, ^58^, ^67^ and that experts and nonexperts found these solutions easy to apply.^22^, ^33^, ^38^ Multiple‐channel solutions, which are easy to apply but with electrodes that are not fixed, such as Emotiv Epoc+^20^ and ANT neuro,^48^ may not be optimal for diagnostic purposes but are useful in situations where it is important to apply the EEG easily. The biggest issues related with devices with low number of channels were related to their visibility,^40^, ^44^, ^45^ the material,^23^ the need for frequent adjustment,^43^, ^44^, ^67^, ^68^ and movement artifacts in the data.^62^ On the other side the use of these solutions is enhanced by the fact that patients and nonexperts needed only a brief training to learn how to apply, fix, or adjust them.^20^, ^26^, ^28^, ^45^, ^49^, ^51^, ^58^ Several solutions described were designed specifically to be discrete,^19^, ^21^, ^27^, ^49^, ^52^ ensure an optimal level of acceptability and usability,^69^ and allow patients to be comfortable.^23^, ^26^, ^28^, ^40^, ^50^ An example of this new approach is a new mobile system in use for ambulatory EEG, the SeerSense (SeerMedical).^70^ Using an innovative water‐soluble electrode adhesive, it permits a quick and easy self‐disconnection and allows patients to have an ambulatory EEG with minimal restriction.

Noninvasive mobile EEG solutions could also have an impact on the economy of health services.^71^ The possibility to automatically detect seizures could decrease the time spent by clinicians reviewing EEG for conventional assessment and can be useful in patients with a low frequency of events.^72^, ^73^ The cost of the systems may be lower compared to conventional in‐hospital scalp EEG and may not be limited by the availability of hospital resources.^21^, ^30^, ^31^, ^32^ Moreover, such systems could be extended to rural areas and populations with limited resources and access to EEG.^21^, ^22^, ^30^

The possibility of easily performing repeated recording at home^30^, ^31^, ^49^, ^59^ may create “patient‐controlled home EEG monitoring,” which has the potential to increase the accuracy of diagnosis, while reducing requirements for hospital‐based monitoring. Mobile solutions also open the possibility for novel applications that are unattainable with conventional systems. For example, a reliable method for detecting and counting seizures using mobile EEG would introduce the opportunity to pre‐emptively modify treatment regimens or plan the optimal timing for diagnostic studies.^74^ Repeated long‐term at‐home recordings could allow seizure forecasting, and identification of seizures pattern and cycles,^75^, ^76^, ^77^, ^78^ which may enable a better understanding of the individual seizure risk over time and improve patients’ quality of life.^79^, ^80^

## 5 CONCLUSION

Our literature review reveals a rapid emergence of noninvasive mobile EEG focused on epilepsy care. Despite promising results, the adoption of these technologies into clinical practice is still limited. Future studies should focus on the assessment of the accuracy, feasibility, and acceptability of such systems in a range of settings. The evidence available is promising, and we believe that new noninvasive mobile EEG has a strong potential to become clinically valuable for the management of people with epilepsy in and outside the hospital.

## AUTHOR CONTRIBUTIONS

AB and VS worked on the literature research, data extraction, data quality assessment, and the manuscript. EB contributed to the organization of the manuscript as a systematic review and data quality assessment. MPR, MPV, PFV, PL, and DP contributed to reviewing the manuscript for publication. All authors have given approval for it to be published.

## Supporting information

Supplementary Material
