# Supplementary material for: Noninvasive mobile EEG as a tool for seizure monitoring and management: A systematic review
Source: Epilepsia. 2022 Mar 27;63(5):1041–63. doi: 10.1111/epi.17220 (PMC9311406; doi:10.1111/epi.17220)
Supplement: Supplementary file 1 — Supplementary Material [file EPI-63-1041-s001.docx]

**Quality assessment – Comparing performance of non-invasive mobile EEG vs standard scalp-EEG**

| QUADAS 2 DOMAIN | QUADAS-2 Description | QUADAS-2 Question | Review Question |
| --- | --- | --- | --- |
| PATIENT SELECTION | Describe method of patient selection  Describe included patients | Are there any concerns that the included patients do not match the review question? | Patients with a diagnosis of epilepsy or patients suspected to have epilepsy and/or seizures requiring EEG for diagnosis |
| INDEX TEST | Describe the index and how it was conducted and interpreted | Are there any concerns that the index, or its interpretation differ from the review question? | Non-Invasive Mobile EEG  Expert visual detection of seizure or EEG abnormalities using non-Invasive Mobile EEG |
| REFERENCE STANDARD | Describe the reference standard and how it was conducted | Are there any concerns that the reference standard does not match the review question? | Visual review of simultaneously acquired standard scalp EEG by expert |

| **Author** | **Participants** | **QUADAS-2 PATIENT SELECTION** | **Non-Invasive Mobile EEG** | **Performance**  **Evaluation/Assessment** | **QUADAS-2**  **INDEX TEST** | **Standard Video-Scalp EEG** | **QUADAS-2 REFERENCE STANDARD** | **Concern about quality** |
| --- | --- | --- | --- | --- | --- | --- | --- | --- |
| Zibrandtsen et al.,^23^ | 15 patients with suspected temporal epilepsy | ☺ | Prototype intra-ear-EEG | Visually compare ictal and interictal abnormalities recorded with ear-EEG and simultaneous scalp-EEG. | ☹ | Mobile EEG recording simultaneously with scalp-EEG | ☺ | The device tested is a prototype and not an FDA or CE approved device available on the market |
| Titgemeyer et al.,^19^ | 22 adults with a diagnosis of epilepsy^ | ☺ | Emotiv EPOC | EEG were visually evaluated by ten independent raters and then compared with respect to the presence of abnormal EEG events (regional slowing, epileptiform potentials, seizure pattern). | ☺ | Mobile EEG recording simultaneously with scalp-EEG | ☺ |  |
| Williams et al., ^21^ | 97 children with epilepsy (mean age 10.3) | ☺ | Custom-made mobile EasyCap with a Smartphone Brain Scanner-2 (SBS2) | EEG were uploaded for remote EEG specialist review and reporting via a web-based reading platform and compared with scalp-EEG. | ? | Mobile EEG not recorded simultaneously with scalp-EEG | ☹ | The measurement of agreement between specialist was relatively low (<0.5).  Recordings took place sequentially so direct comparison between simultaneous EEG data was not possible |
| McKenzie et al.,^22^ | 205 patients with epilepsy | ☺ | Custom-made mobile EasyCap with a smartphone Brain Scanner-2 (SBS2) | EEG were uploaded for remote EEG specialist review and reporting via a web-based reading platform and compared with scalp-EEG | ☺ | Mobile EEG not recorded simultaneously with scalp-EEG. | ☹ | Recordings took place sequentially so direct comparison between simultaneous EEG data was not possible |
| Carvalho et al.,^24^ | 38 patients with continuous spike-wave of sleep (CSWS) | ☺ | Prototype bipolar behind the ear EEG (Neury-2) | Demonstrate the clinical value of repeated spike index assessments using a wearable EEG device. Visual inspection of spike quantification. | ☹ | Mobile EEG recording simultaneously with scalp-EEG | ☺ | The device tested is a prototype and not an FDA or CE approved device available on the market |
| Frankel et al., ^27^ | 40 adults with epilepsy^ | ☺ | Epilog | Determine how accurate epileptologists are at remotely reviewing Epilog sensor EEG. Determine which seizure types can be electrographically and visually counted from the mobile EEG device. | ☺ | Mobile EEG recording simultaneously with scalp-EEG | ☺ |  |
| Swinnen et al., ^25^ | 12 adult and children with epilepsy | ☺ | Sensor Dot (Byteflies) | Determine how accurate epileptologists are at reviewing and identifying absence seizure using Sensor Dot EEG. | ☺ | Mobile EEG recording simultaneously with scalp-EEG | ☺ |  |

**Quality Assessment - Quality of the EEG data collected**

| QUADAS 2 DOMAIN | QUADAS-2 Description | QUADAS-2 Question | Review Question |
| --- | --- | --- | --- |
| PATIENT SELECTION | Describe method of patient selection  Describe included patients | Are there any concerns that the included patients do not match the review question? | Patients with a diagnosis of epilepsy or patients suspected to have epilepsy and/or seizures requiring EEG for diagnosis |
| INDEX TEST | Describe the index and how it was conducted and interpreted | Are there any concern that the index, or its interpretation differ from the review question? | Non-Invasive Mobile EEG  Quality of the signal recorded with non-invasive mobile EEG and compared to standard scalp-EEG (Correlation Coefficient) |
| REFERENCE STANDARD | Describe the reference standard and how it was conducted | Are there any concern that the reference standard does not match the review question? | Standard Video Scalp-EEG |

| **Author** | **Participants** | **QUADAS-2 PATIENT SELECTION** | **Non-Invasive Mobile EEG** | **Performance**  **Quality Assessment** | **QUADAS-2**  **INDEX TEST** | **Standard Video-Scalp EEG** | **QUADAS-2 REFERENCE STANDARD** | **Concern about quality** |
| --- | --- | --- | --- | --- | --- | --- | --- | --- |
| Zibrandtsen et al.,^23^ | 15 patients with suspected temporal epilepsy | ☺ | Prototype intra-ear-EEG | Quantify similarities between data collected from the two solutions. | ☺ | Mobile EEG recording simultaneously with scalp-EEG | ☺ | The device tested is a prototype and not an FDA or CE approved device available on the market |
| Kutafina et al.,^29^ | 22 adults with a diagnosis of epilepsy^ | ☺ | Emotiv EPOC | Develop a computer-based analysis pipeline, to compare the EEG signal acquired by a mobile EEG device to video scalp EEG. | ☺ | Mobile EEG recording simultaneously with scalp-EEG | ☺ |  |
| Kamousi et al.,^35^ | 22 patients with altered mental status and suspected non-convulsive and subclinical seizures. | ☺ | Rapid-EEG by Ceribell (8 channel portable solution) | Evaluating the signal quality of EEG waveforms acquired with the tested rapid response EEG system in comparison to conventional  clinical EEG systems in laboratory as well as clinical ICU settings. | ☺ | Mobile EEG recorded prior to conventional scalp-EEG | ☹ | The EEG were not recorded simultaneously, preventing comparison of power spectrum or events happening simultaneously. |
| Meyer et al., ^33^ | 52 patients with vigilance reduction [(21%) with epileptic seizure or status] | ☺ | CerebAir EEG headset | Compare data quality of mobile solution and standard scalp EEG. | ☺ | Mobile EEG recording simultaneously with scalp-EEG | ☺ |  |
| Sokolov et al.,^30^ | 149 patients with epilepsy | ☺ | Custom-made mobile EasyCap with a Smartphone Brain Scanner-2 (SBS2) | Assess the quality and reproducibility of the EEG output recorded with a low-cost mobile EEG device. | ☹ | Mobile EEG not recorded simultaneously with scalp-EEG.  Mobile EEG recorded two times on the same subjects. | ☹ | Conventional scalp EEG was not collected, preventing any assessment of signal quality in comparison to conventional scalp EEG. |

**Quality Assessment - Diagnostic value of EEG signal recorded via non-invasive mobile EEG in the intensive care unit (ICU) and emergency department (EM)**

| QUADAS 2 DOMAIN | QUADAS-2 Description | QUADAS-2 Question | Review Question |
| --- | --- | --- | --- |
| PATIENT SELECTION | Describe method of patient selection  Describe included patients | Are there any concerns that the included patients do not match the review question? | Patients with a diagnosis of epilepsy or patients suspected to have epilepsy and/or seizures requiring EEG for diagnosis |
| INDEX TEST | Describe the index and how it was conducted and interpreted | Are there any concern that the index, or its interpretation differ from the review question? | Non-Invasive Mobile EEG  Diagnostic value of Non-Invasive Mobile EEG signal |
| REFERENCE STANDARD | Describe the reference standard and how it was conducted | Are there any concern that the reference standard does not match the review question? | Standard Scalp-EEG |

| **Author** | **Participants** | **QUADAS-2 PATIENT SELECTION** | **Non-Invasive Mobile EEG** | **Aim of the study** | **QUADAS-2**  **INDEX TEST** | **Standard Video-Scalp EEG** | **QUADAS-2 REFERENCE STANDARD** | **Concern about quality** |
| --- | --- | --- | --- | --- | --- | --- | --- | --- |
| Meyer et al.,^33^ | 52 patients with vigilance reduction [(21%) with epileptic seizure or status] | ☺ | CerebAir EEG headset  (AE-120A EEG Headset) | Compare seizure detection performance of clinicians using a mobile solution and a standard scalp EEG. | ? | Mobile EEG recording performed at a different time and not simultaneously with scalp-EEG | ☹ | Only 47 of 52 patients received both mobile EEG and conventional scalp EEG. Scalp EEG was performed at a different time, typically several hours apart from mobile EEG recording. |
| Egawa et al.,^34^ | 55 with altered mental status  [6 of them (12%) with epilepsy diagnosis] | ☹ | CerebAir EEG headset  (AE-120A EEG Headset) | Examine the diagnostic accuracy of Cerebair EEG monitoring in detecting abnormal EEG patterns and NCSE in patients with altered mental status (AMS) with unknown aetiology. | ☺ | Mobile EEG recording not simultaneously but before the scalp-EEG | ☹ | A relatively small number of patients with relevant EEG abnormalities were included in this study; hence, the results require confirmation in a larger cohort. |
| Vespa et al.,^38^ | 164 patients with encephalopathy and suspected non-convulsive and subclinical seizures (32% witnessed seizure) | ☺ | Rapid-EEG by Ceribell (8 channel portable solution) | To measure the seizure diagnosis accuracy of clinicians using the Ceribell rapid response in the ICU | ☹ | Mobile EEG not recorded simultaneously but before with scalp-EEG | ☹ | First, the evaluation was limited to theoretical diagnostic and therapeutic decision making and, as such, evaluation of actual treatment decisions based on Rapid-EEG data and patients’ clinical outcomes were not studied.  The current study measured the impact of Rapid-EEG system used at the bedside by residents, fellows, and attending physicians who had varying degrees of neurology training. |

**Quality Assessment – The application of Automatic Detection algorithms to non-invasive mobile EEG recordings**

| QUADAS 2 DOMAIN | QUADAS-2 Description | QUADAS-2 Question | Review Question |
| --- | --- | --- | --- |
| PATIENT SELECTION | Describe method of patient selection  Describe included patients | Are there any concerns that the included patients do not match the review question? | Patients with a diagnosis of epilepsy or patients suspected to have epilepsy and/or seizures requiring EEG for diagnosis |
| INDEX TEST | Describe the index and how it was conducted and interpreted | Are there any concern that the index, or its interpretation differ from the review question? | Non-Invasive Mobile EEG  Automatic detection algorithm performance (Sensitivity & Specificity) |
| REFERENCE STANDARD | Describe the reference standard and how it was conducted | Are there any concern that the reference standard does not match the review question? | Manual and visual detection by expert using Standard Scalp-EEG |

| **Author** | **Participants** | **QUADAS-2 PATIENT SELECTION** | **Non-Invasive Mobile EEG** | **Performance**  **Quality** | **QUADAS-2**  **INDEX TEST** | **Standard Video-Scalp EEG** | **QUADAS-2 REFERENCE STANDARD** | **Concern about quality** |
| --- | --- | --- | --- | --- | --- | --- | --- | --- |
| Kjaer et al.,^40^ | 6 children with suspected epilepsy (aged 5-16) | ☺ | Mobile EEG recorder (Actiwave, CamNtech Ltd) connected with 3 electrodes | Evaluate how well an automatic seizure detection algorithm can identify absences. | ☺ | Mobile EEG recording simultaneously with scalp-EEG and then at home without scalp EEG. | ☺ |  |
| Swinnen et al.,^25^ | 12 adult and children with epilepsy | ☺ | Sensor Dot EEG  (Byteflies) | Develop a sensitive patient-specific absence seizure detection algorithm to reduce the review time of the recordings. | ☺ | Mobile EEG recording simultaneously with scalp-EEG | ☺ |  |
| Frankel et al.,^41^ | 20 adults with epilepsy and seizure during hospitalization | ☺ | Epilog Epitel | Determine accuracy of expert and automated seizure detection on Epilog recordings. | ☺ | Mobile EEG recording simultaneously with scalp-EEG | ☺ | The number of events used for the evaluation of  the diagnostic accuracy of manual seizure detection (31 epochs with ictal events and 83 non-ictal) was not balanced  . |
| Kamousi et al.,^42^ | 353 adults who underwent monitoring with Rapid EEG Ceribell | ☺ | Rapid-EEG by Ceribell (8 channel portable solution) | To test the performance of a machine learning method that generates bedside alerts for possible status epilepticus (seizure > 5mins)  and measures in real time the burden of seizure activity  [(10%) at least 30 sec,  (50%) at least 2.5 mins,  (90%) > 5mins seizure activity] | ☹ | Mobile EEG not recorded simultaneously but before scalp-EEG | ? | The cohort contained a relatively low number of status epilepticus cases (nine out of 353 EEGs) which affects the reliability of our sensitivity estimate. A larger cohort of Rapid-EEG data would be helpful to address this. |

**Quality Assessment – The tolerance, usability and acceptability of non-invasive mobile EEG system**

| QUADAS 2 DOMAIN | QUADAS-2 Description | QUADAS-2 Question | Review Question |
| --- | --- | --- | --- |
| PATIENT SELECTION | Describe method of patient selection  Describe included patients | Are there any concerns that the included patients do not match the review question? | Patients with a diagnosis of epilepsy or patients suspected to have epilepsy and/or seizures requiring EEG for diagnosis |
| INDEX TEST | Describe the index and how it was conducted and interpreted | Are there any concern that the index, or its interpretation differ from the review question? | Acceptability, Tolerability and Usability score from patients or experts using the non-invasive mobile EEG. |
| REFERENCE STANDARD | Describe the reference standard and how it was conducted | Are there any concern that the reference standard does not match the review question? | Data collected using a standardized questionnaire or semi/structured interview |

| **Author** | **Participants** | **QUADAS-2 PATIENT SELECTION** | **Non-Invasive Mobile EEG** | **Aim of the study** | **QUADAS-2**  **INDEX TEST** | **Standard Survey or Interview** | **QUADAS-2 REFERENCE STANDARD** | **Concern about quality** |
| --- | --- | --- | --- | --- | --- | --- | --- | --- |
| Kjaer et al.,^40^ | 6 children with suspected epilepsy (aged 5-16) | ☺ | Mobile EEG recorder (Actiwave, CamNtech Ltd) connected with 3 electrodes | Evaluate how easily outpatients can be monitored with a mobile behind the ear solution. | ☺ | N/P | ☹ | Experience evaluated without the use of a standardized questionnaire or interview. |
| Simblett et al.,^44^ | 8 adults with a diagnosis of epilepsy | ☺ | Epilog alongside other wearables | Assess the first-hand experiences of people with epilepsy using wearable devices and understand how acceptable and easy they were to use. | ☺ | Semi-Structured Interview | ☺ |  |
| Bruno et al.,^43^ | 12 adults with a diagnosis of epilepsy | ☺ | Epilog alongside other wearables | Evaluate the experience of using wearables device during video-EEG in patients with epilepsy | ☺ | Modified version of a validated questionnaires  [TAM-FF Technology Acceptance Model Fast Form] | ☺ |  |
| Carvalho et al.,^24^ | 38 patients with continuous spike-wave of sleep (CSWS) | ☺ | Prototype bipolar behind the ear EEG (Neury) | N/P | ☹ | N/P | ☹ | They reported information about tolerability without using any standardized survey or questionnaire for the assessment. Main aim of the study was not to evaluate acceptability or usability of mobile EEG. |
| McKenzie et al.,^22^ | Patients and expert/non expert using the non-invasive mobile EEG | ☹ | Custom-made mobile EasyCap with a smartphone Brain Scanner-2 (SBS2) | N/P | ☹ | N/P | ☹ | They reported information about usability tolerability without using any standardized survey or questionnaire for the assessment. Main aim of the study was not to evaluate acceptability or usability of mobile EEG. |
| Meyer et al.,^33^ | Nurses in the ICU applying the non-invasive mobile EEG | ☹ | CerebAir | N/P | ☹ | N/P | ☹ | They reported acceptability from nurse working in the ICU without assessing it using any standardized survey or questionnaire for the assessment. Main aim of the study was not to evaluate acceptability or usability of mobile EEG. |
| Vespa et al.,^38^ | Technician in the ICU applying the non-invasive mobile EEG | ☺ | Ceribell | Evaluate how easy to use is the Ceribell EEG Cap in the ICU | ☺ | A 5 items Likert scale | ☺ |  |
| Zibrandtsen et al.,^23^ | 15 patients with suspected temporal epilepsy | ☺ | Prototype intra-ear-EEG | N/P | ☹ | N/P | ☹ | They reported the main practical issues related with the use of the ear-EEG without using a standardized survey or interview |
| Olsen et al.,^45^ | 9 outpatients with epilepsy | ☺ | Portable EEG amplifier with 2 channels | To explore the experiences of people with epilepsy using wearables for home seizure monitoring. | ☹ | Two structured interviews were performed (One before wearing the device and the second one after wearing the device) | ☺ | The wearable was not an FDA or CE marked device.  It was described in the manuscript, but some technical information was omitted. |

N/P = Not performed. The aim of the study was not to assess quality or usability of the non-invasive device. The study did not assess the acceptability or usability of the device using a standardized questionnaire or semi-structured/structured interview.

Low Risk High Risk Potential Risk

**SEARCH STRATEGY**

**Database:**
Embase
Ovid MEDLINE(R) ALL

Global Health
APA PsycInfo

| **#** | **Query** | **Results from Jan Week 3 2022** |
| --- | --- | --- |
| 1 | **((Ear or wireless or Bluetooth or portable or mobile or wearable or smartphone or rapid response) and (EEG or electroencephalograp*) and (epilep* or seizur*)).mp. [mp=ti, ab, hw, tn, ot, dm, mf, dv, kf, fx, dq, nm, ox, px, rx, an, ui, sy, bt, id, cc, tc, tm]** | 1,558 |
| 3 | **limit 1 to english language** | 1,462 |
| 4 | **limit 2 to yr="2000 -Current"** | 1,325 |
| 5 | **remove duplicates from 3** | 927 |
